# From Stage to Systems: Integrating Standard Laboratory Markers into Bladder Cancer Prognostic Models

**DOI:** 10.3390/diagnostics16213486

**Published:** 2026-10-24

**Authors:** Said el Bouhaddani, Ahmed Albashri, Osama Almohammadi, Muath Alamri, Lujain Alqarni, Sahar Aiman Almaimani, Sadeel Abdullah Alqattan, Shahad Alrbaie, Fatmah Fallatah, Reema Safar Alamri, Wed Bakudam, Manal Alghamdi, Khaled Albarakati, Hadeel Alshangiti, Nada Albarakati

**Affiliations:** 1 Department of Population Health Research, King Abdullah International Medical Research Center, Jeddah 22384, Saudi Arabia; elbouhaddanis@kaimrc.med.sa; 2 Department of Blood and Cancer Research, King Abdullah International Medical Research Center/King Saud Bin Abdulaziz University for Health Sciences, Ministry of National Guard Health Affairs, Jeddah 21423, Saudi Arabia; 3 College of Medicine, University of Jeddah, Jeddah 21589, Saudi Arabia; 4 Faculty of Medicine, Ibn Sina National College for Medical Studies, Jeddah 22421, Saudi Arabia; 5 Faculty of Medicine, King Abdulaziz University, Jeddah 21589, Saudi Arabia; 6 School of Medicine, University College Dublin, D04 V1W8 Dublin, Ireland; 7 Department of Blood and Cancer Research, King Abdullah International Medical Research Center, Jeddah 21423, Saudi Arabia

**Keywords:** bladder cancer, machine learning, prognostic modeling, routine laboratory markers

## Abstract

**Background/Objectives**: Bladder cancer (BC) remains a globally prevalent malignancy with significant clinical heterogeneity. While the TNM staging system is the diagnostic standard, its ability to provide individualized, comprehensive prognostic insights is limited. This study evaluates whether integrating routine laboratory markers into machine learning (ML) frameworks can enhance mortality risk prediction beyond traditional staging. **Methods**: Routine electronic health records from 337 adult patients with confirmed BC (2010–2023) were analyzed. Six ML architectures were evaluated for mortality risk prediction: logistic regression (GLM), elastic net logistic regression (GLMNET), generalized additive models (GAM), extreme gradient boosting (XGBoost), gradient boosting machines (GBM), and support vector machines (SVM). **Results**: All models demonstrated stable performance, with the SVM achieving the highest pooled AUC (0.679, 95% CI 0.57–0.77). Feature importance analyses identified tumor grade and routine blood markers, specifically platelet count, bicarbonate (CO_2_), and random glucose, as the highest-ranking predictors, demonstrating a greater relative feature weight than individual traditional anatomical staging parameters within the models. **Conclusions**: Integrating standard hematologic and metabolic panels into ML models offers a potential, cost-effective avenue to augment risk stratification, warranting further external validation before clinical implementation.

## 1. Introduction

Bladder cancer (BC) is the tenth most commonly diagnosed cancer worldwide and remains a major cause of cancer-related morbidity, mortality, and healthcare expenditure [1,2]. Its clinical course is highly variable: many patients present with non-muscle-invasive disease and exhibit more favorable survival profiles, whereas those who develop muscle invasion or metastases face a sharp decline in long-term outcomes despite advances in surgery, systemic therapy, and immuno-oncology [1,3,4]. This biological and clinical heterogeneity makes accurate risk stratification central to treatment planning and follow-up.

In routine practice, prognosis is still guided primarily by anatomical staging (TNM), histologic grade, and a limited set of clinicopathological factors [1,5]. Although these tools are well established, patients with similar stage and grade often experience markedly different trajectories, highlighting the limitations of morphology-based systems for individualized prediction [4,5,6]. In both non-muscle-invasive and muscle-invasive disease, misclassification of risk can lead to overtreatment in some patients and delayed or insufficient therapy in others [7,8,9]. There is therefore a pressing need for complementary prognostic approaches that capture systemic host factors and tumor biology beyond anatomy alone.

Over the past decade, artificial intelligence (AI) and machine learning (ML) have been rapidly integrated into BC research across multiple data modalities, including histopathology, radiomics, and multi-omics [2,7,10,11]. ML-based signatures derived from whole-slide images, CT and MRI radiomics, genomic and transcriptomic profiles, and exosome-related genes have repeatedly shown superior diagnostic and prognostic performance compared with conventional staging and grading, often improving discrimination indices and reclassifying risk beyond TNM (UICC/AJCC) systems [6,9,12,13]. However, many of these models depend on specialized imaging platforms, complex multi-omics pipelines, or extensive bioinformatics infrastructure, which can be expensive, technically demanding, and not widely accessible in everyday practice [2,10,14]. As a result, their immediate translational impact remains limited.

In contrast, standard clinicopathological data and routine laboratory tests such as complete blood counts and basic metabolic panels are inexpensive, universally available, and reflect systemic processes including inflammation, nutritional status, and metabolic stress, all of which are increasingly recognized as important determinants of cancer outcomes [8,15,16,17]. Emerging work in bladder and other solid tumors suggests that combining these systemic markers with established clinical variables can meaningfully improve survival prediction, sometimes outperforming models based on staging alone [6,7,9,18]. Yet, few studies have rigorously compared the prognostic contribution of common hematologic and metabolic parameters against TNM stage within modern, interpretable ML frameworks, particularly in clinically realistic cohorts that respect events-per-predictor constraints.

The present study addresses this gap by focusing on prognostication using data already available in routine clinical practice. The overarching aim is twofold: first, to identify which standard blood markers carry independent prognostic information; and second, to evaluate whether these laboratory markers can enhance mortality risk prediction beyond traditional anatomical staging. To this end, multiple machine learning architectures, including linear, additive, tree-based, and kernel-based models, were trained and validated. Ultimately, this work demonstrates the capacity of ML to capture latent patterns in common hematologic and metabolic measures, highlighting their potential to eventually complement personalized surveillance and patient counseling.

## 2. Materials and Methods

We follow the STROBE and TRIPOD reporting guidelines for observational and prediction model studies to structure the manuscript.

### 2.1. Study Cohort

We conducted a retrospective cohort study using routinely collected electronic health records from the National Guard Health Affairs (NGHA) BESTCare system, covering the period from July 2010 until June 2023. This system operates within the Ministry of the National Guard Health Affairs (MNGHA) in Jeddah, Saudi Arabia, a multi-specialty, tertiary care academic medical center that serves National Guard personnel, their dependents, and referred civilian patients from across the Western Region. The study protocol was approved by the Institutional Review Board of the King Abdullah International Medical Research Center (KAIMRC). Informed consent was waived due to the retrospective design.

The source population comprised all adult patients with a histologically confirmed diagnosis of BC recorded in the system. Eligible individuals were aged ≥18 years at the time of diagnosis. Cohort entry was defined as the date of histopathological confirmation of BC. This date was considered the baseline for prediction. Only information available prior to initiation of chemotherapy or radiotherapy was used. Patients with unknown survival status were excluded. All eligible patients were included in the analysis. No formal sample size calculation was performed. The final dataset consisted of *N* = 337 patients. The study follow-up window closed on 30 June 2023. Within this finalized dataset, the median overall follow-up duration was 21 months (Interquartile Range [IQR]: 7–51 months), which provided a baseline timeline to capture subsequent mortality events.

### 2.2. Variables

The primary outcome was a binary indicator of all-cause mortality during follow-up. Patient vital status was ascertained and verified directly through institutional Electronic Health Records (EHR). Mortality was coded as 1 if death occurred at any time between diagnosis and end of follow-up, and 0 otherwise. Predictors further included demographic characteristics (age at diagnosis, sex), tumor-related characteristics (tumor grade, stage, morphology, muscle invasiveness, metastatic status at presentation, papillary tumor, primary lymph node presentation, recurrence after surgery), and routinely measured laboratory parameters.

Tumor grade was categorized as low (Grade I) versus high grade (Grade II and III). Tumor stage was dichotomized based on numerical pathological T-codes: early stage (coded 1) encompassed T0, Ta, T1, and Tis, while late stage (coded 2) encompassed T2 (including T2a, T2b), T3 (including T3a, T3b), and T4 (including T4a, T4b). Separately, muscle invasiveness was captured as a distinct variable to differentiate Non-Muscle-Invasive (NMIBC) from Muscle-Invasive Bladder Cancer (MIBC), thereby accurately accommodating high-risk microinvasions or clinical discrepancies regardless of a baseline T1 designation. Morphology was classified into transitional cell carcinoma, squamous cell carcinoma, and adenocarcinoma. Laboratory values were extracted from structured EHR fields. For patients with multiple laboratory measurements prior to treatment, the value closest to the date of diagnosis was selected.

Continuous predictors were analyzed on their original scale. No categorization of continuous variables was performed. Where required by the algorithm, predictors were standardized.

### 2.3. Missing Data

Predictors with missingness ≥ 80% were excluded prior to modeling. Nine laboratory variables met this criterion and were removed. For the remaining predictors, the average proportion of missing data was 37.5%. In total, 22 predictors were used. No additional pre-modeling feature selection was performed.

Missing values were handled using multiple imputation by chained equations under a missing-at-random assumption. As a diagnostic check of this assumption, Fisher’s exact tests were performed to evaluate associations between missingness indicators and age, sex, and outcome after multiple testing correction with Holm. Forty imputed datasets were generated, which is comparable to the percentage of missing data. Each imputation used 50 iterations per chain. All predictors and the outcome variable were included in the imputation model. Continuous variables were imputed using predictive mean matching. Binary and categorical variables were imputed using logistic and multinomial regression models. To assess potential collinearity after imputation, pairwise correlations were computed within each imputed dataset and summarized across imputations.

### 2.4. Prediction Models

Six prediction models were considered to estimate mortality risk during follow-up: logistic regression (GLM), elastic net logistic regression (GLMNET), generalized additive models (GAM), extreme gradient boosting (XGBoost), gradient boosting machines (GBM), and support vector machines (SVM) with radial basis function kernels. These models represent linear, additive, tree, and kernel-based approaches to predict risk.

Logistic regression models included all predictors without interaction terms. Elastic net regression combined L1 and L2 penalties to allow shrinkage and variable selection. For generalized additive models, penalized smooth terms were included for continuous predictors to allow non-linear effects. Smoothing parameters were estimated by restricted maximum likelihood.

For tree-based models, model complexity was controlled through tuning of tree depth, learning rate, and related hyperparameters. For the support vector machine, cost and kernel parameters were tuned. Hyperparameter tuning was performed using stratified 10-fold cross-validation within each imputed dataset. The area under the ROC curve (AUC) was used as the objective function. The complete hyperparameter tuning grids are given in the Supplementary Materials (Table S5).

Cross-validation was performed within each imputed dataset to avoid information leakage. Final performance estimates were pooled across imputations using the *psfmi* R 4.5.3 package. Final predicted probabilities are calculated on the left-out folds and aggregated across imputations using the median.

### 2.5. Model Evaluation and Interpretation

Model discrimination was assessed using the cross-validated AUC, and pooled across imputations. We denote this by the pooled (cross-validated) AUC. Classification performance was additionally evaluated. We calculated the Youden index to achieve a balance between sensitivity and specificity. Note that ideally the threshold is set by the intended clinical use rather than fixed in advance. Sensitivity, specificity, positive predictive value, negative predictive value, and F1 score were also based on cross-validated metrics. For visualizing the ROC curves, the predicted probabilities on the left-out folds were taken and pooled, with which the pooled cross-validation-based ROC curves are constructed. The area under this pooled cross-validation-based ROC is referred to as a ‘recomputed AUC’ based on the pooled probability predictions. Note that the pooled (cross-validated) AUC is the main model evaluation metric.

Calibration was assessed using calibration plots comparing predicted and observed risks. Logistic calibration curves were fitted to evaluate correspondence between observed and predicted risk. Clinical utility was explored using decision curve analysis, estimating standardized net benefit across a range of threshold probabilities. Both calculations are based on left-out fold predictions.

To interpret model behavior, variable importance (VIP) measures and SHapley Additive exPlanations (SHAP) values were computed. Importance scores were normalized and averaged across imputations and across models. SHAP values quantified the average absolute contribution of each predictor to individual predictions.

## 3. Results

### 3.1. Descriptive Results

A total of 337 adults with histologically confirmed BC met the inclusion criteria and were included in the final cohort. Of these, 228 patients (67.6%) died during follow-up. The majority of patients were male (85%). Median age at diagnosis was 64 years (interquartile range 55 to 72). Median overall survival was 21 months. In total, 22 predictors were used.

Baseline characteristics of the study population are presented in Table 1. High-grade tumors were common (59%), and 23% of patients had muscle-invasive disease at baseline. Metastatic disease at presentation was observed in 14% of cases. Transitional cell carcinoma was the predominant morphology and accounted for more than 90% of tumors.

Nine predictors were excluded prior to modeling due to missingness exceeding 80%. For the remaining variables, the average proportion of missing data was 37.5%. A Fisher exact test revealed no significant associations between the missingness indicator and age, sex, or outcome, except for MPV, which was associated with the outcome (adjusted *p* = 0.015). See the Supplementary Materials Section “Missingness analyses and demographic patterns” for the complete analysis. After multiple imputation, correlations between predictors were evaluated across the 40 imputed datasets. The distribution of absolute correlations showed generally low dependence, with a median absolute correlation of 0.080 and a mean of 0.12 (IQR = 0.041–0.144). This suggests that most predictor pairs were not strongly collinear.

### 3.2. Prediction Modeling Results

The pooled cross-validated performance of the prediction models is summarized in Table 2. Discrimination was modest and broadly comparable across the six modeling approaches. Pooled cross-validated AUC values ranged from 0.64 to 0.68. Support vector machines achieved the highest pooled AUC (0.679, 95% CI 0.57 to 0.77), followed by elastic net regression (0.671, 95% CI 0.55 to 0.77) and logistic regression and extreme gradient boosting with similar estimates (0.662 to 0.663). The generalized additive model showed slightly lower discrimination (0.653, 95% CI 0.54 to 0.75), and gradient boosting machines had the lowest AUC (0.639, 95% CI 0.52 to 0.74). Overall, confidence intervals were mostly overlapping across all methods, indicating that differences between models were not significant.

Pooled cross-validated sensitivity and specificity were relatively balanced across models. Sensitivity ranged from 0.610 (95% CI 0.35 to 0.82) for XGBoost to 0.705 (95% CI 0.43 to 0.88) for GLMNET, while specificity ranged from 0.601 (95% CI 0.36 to 0.80) for support vector machines to 0.680 (95% CI 0.42 to 0.86) for XGBoost. Positive predictive values were similar across models, ranging from 0.783 to 0.799, whereas negative predictive values were lower and ranged from 0.449 to 0.494. F1 scores ranged from 0.683 (95% CI 0.50 to 0.82) for XGBoost to 0.736 (95% CI 0.62 to 0.83) for support vector machines.

**Figure 1:**
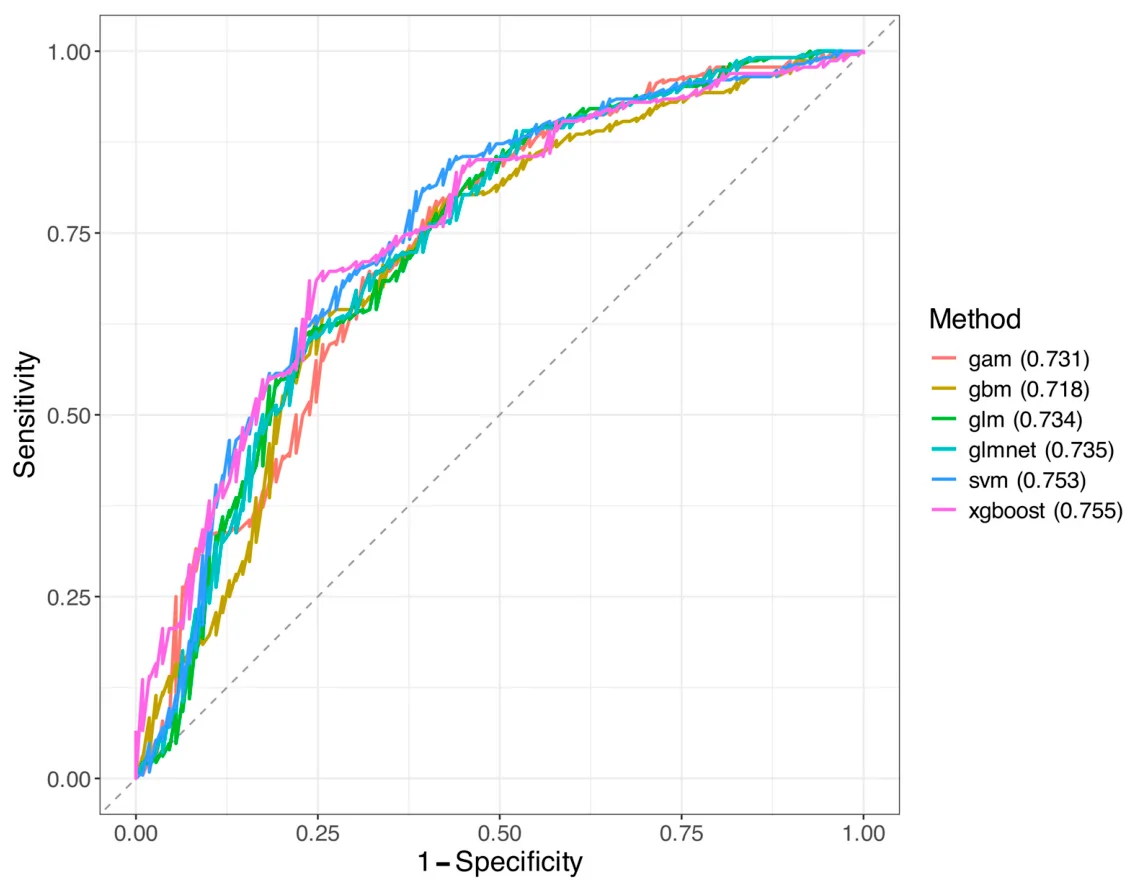
Pooled cross-validation-based ROC curves for six mortality prediction models (glm, glmnet, gam, gbm, svm, xgboost). Curves show sensitivity versus 1-specificity across thresholds; the dashed diagonal line indicates no discrimination. Legend values denote recomputed AUCs based on the ROCs, not the main pooled cross-validated AUC metrics.

Receiver operating characteristic curves are shown in Figure 1. The pooled cross-validation-based ROC curves show substantial overlap across models over most of the false positive rate range. Note that the AUC values in this figure are recomputed after pooling the predictions and slightly overestimate the performance, whereas the pooled cross-validated metrics in Table 2 are to be taken as final performance metrics. Visually, differences were negligible and not consistent across all thresholds. Overall, discrimination differs only slightly between approaches.

### 3.3. Calibration and Clinical Utility

**Figure 2:**
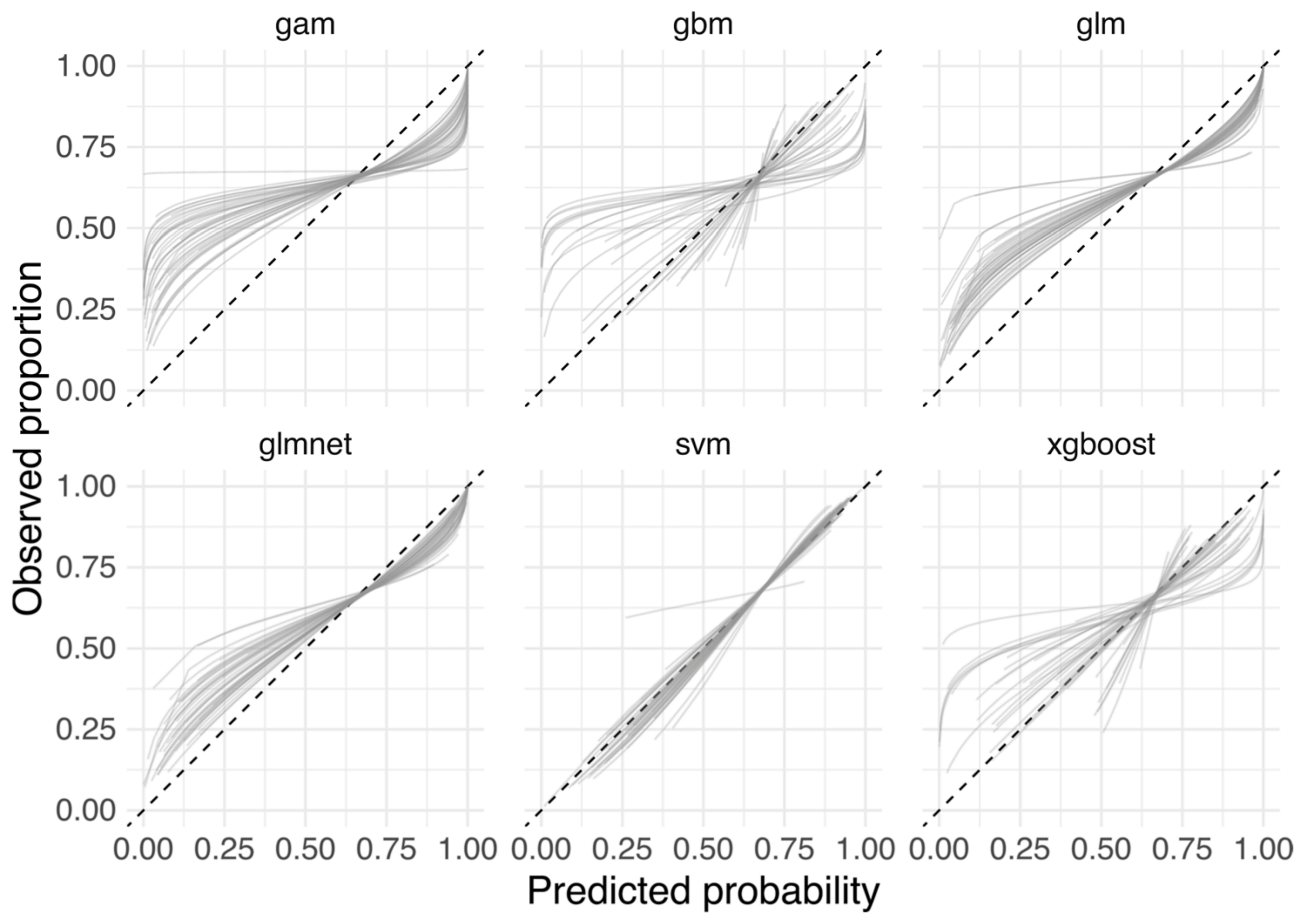
Cross-validation-based calibration plots for six prediction models. The x-axis shows predicted mortality probability, the y-axis the observed event proportion. The dashed line indicates perfect calibration. Gray curves represent imputation-specific calibration curves.

Cross-validation-based calibration curves are shown in Figure 2. Overall, there was a tendency to overestimate low and high risks. Support vector machines showed good overall alignment, with relatively limited divergence from the identity line, while the linear and additive models were slightly miscalibrated. For the more flexible tree-based methods, calibration was less stable across imputations. Extreme gradient boosting and gradient boosting machines showed larger spread between imputation-specific curves and overall deviations from the identity line.

**Figure 3:**
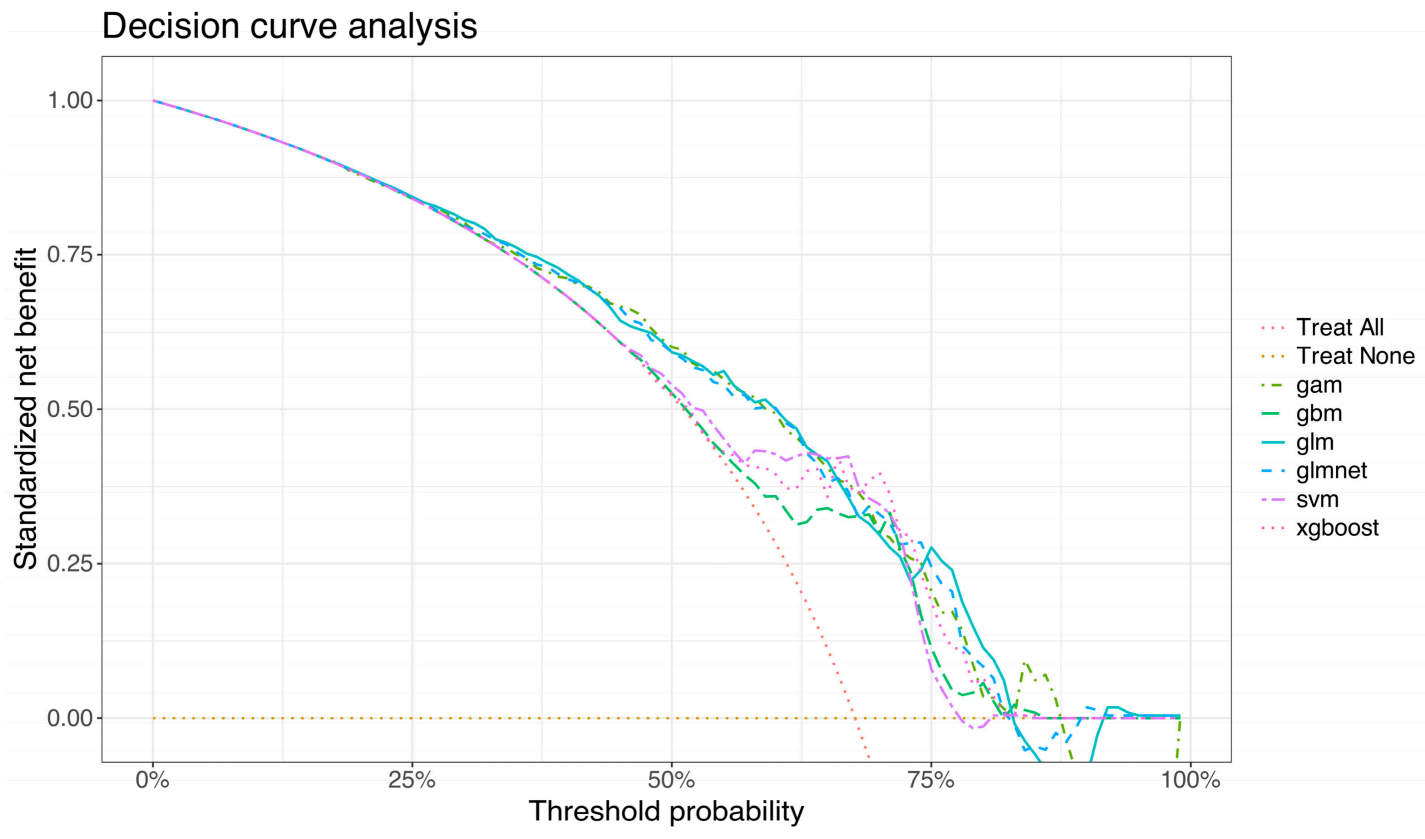
Cross-validation-based decision curve analysis comparing standardized net benefit across threshold probabilities for six prediction models. The treat-all and treat-none strategies are shown as reference. Higher curves indicate greater clinical utility at the corresponding risk threshold.

Cross-validation-based decision curve analysis for the pooled predictions is presented in Figure 3. Since there is no treatment, the ‘treat’ label should be interpreted as predicting all resp. none as deceased. All models showed positive standardized net benefit compared with a predict-none strategy across a broad range of threshold probabilities. The net benefit curves were largely overlapping, indicating only small differences in clinical utility between modeling approaches. Relative to a predict-all strategy, the models provided higher net benefit, especially between 70% and 80%. At higher thresholds, net benefit decreased for all approaches and converged toward zero, with some instability in the tails where few patients are classified as high risk. Overall, the figure suggests similar apparent clinical utility across methods with a slight advantage over the predict-all and none strategies.

### 3.4. Predictor Importance and Robustness

Variable importance measures and SHAP analyses are summarized in Table 3 (see Table S1 in Supplementary Materials for all predictors). Across the modeling approaches, tumor grade was consistently ranked as the most influential predictor. It had the highest overall VIP score (60.51) and also the largest mean absolute SHAP contribution when averaged across methods (mean absolute SHAP 0.1236). Platelet count was also deemed predictive (VIP 49.48; SHAP 0.1191). The top five further included bicarbonate (CO_2_), random glucose, red blood cell count, and MCV. The predictor MPV, whose missingness was significantly associated with the outcome, was not ranked as a top predictor.

Note that differences between the two approaches are expected, because VIP reflects global model dependence while SHAP reflects average local contribution, and rankings can shift depending on whether non-linear interactions or correlated predictors are used by the model.

**Figure 4:**
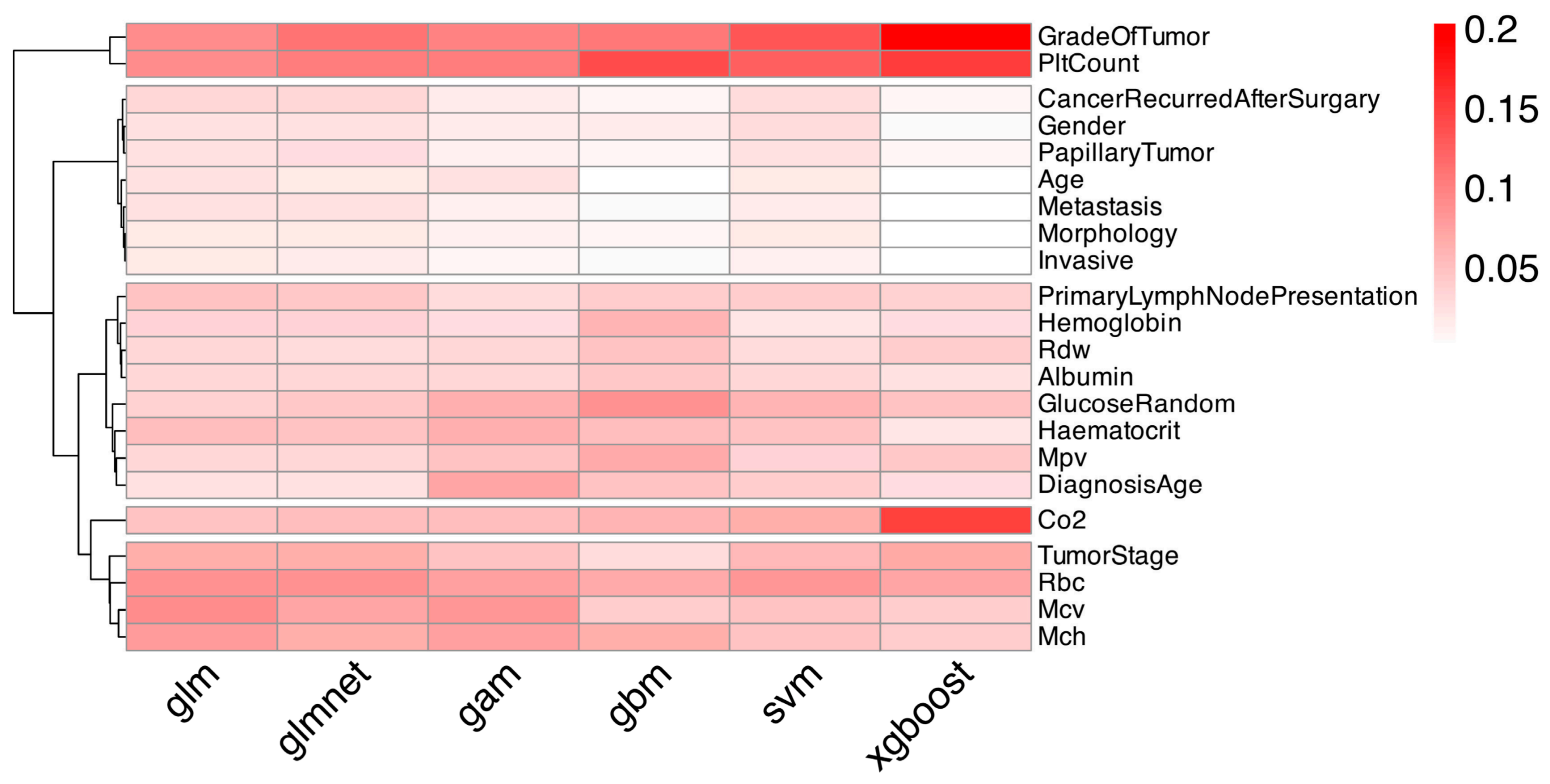
Heatmap of normalized mean absolute SHAP values per predictor and modeling approach. Rows represent predictors and columns represent models; the overall mean across methods is added in the last column. Darker shading indicates larger mean absolute SHAP (stronger average contribution to predictions). Rows are hierarchically clustered (Ward.D2) to group predictors with similar importance patterns across models.

Figure 4 shows a heatmap of mean absolute SHAP values per predictor (rows) and modeling approach (columns). Predictors are clustered to reveal consistently high importance across methods. Tumor grade and platelet count are the dominant features overall, with consistently high contributions across all algorithms. Several laboratory markers related to hematology and metabolism contribute in a more heterogeneous way. For example, CO_2_ shows a pronounced contribution in XGBoost compared with other models, which might indicate that the signal was partly non-linearly associated with the outcome. SHAP values per individual prediction are shown in Figure S1 (Supplementary Materials).

Model performance patterns and predictor rankings were stable across models. No single algorithm demonstrated clear superiority. Linear and non-linear approaches achieved comparable discrimination and calibration, which suggests that the prognostic signal is present in the predictor set itself rather than being driven by one specific modeling framework.

To directly assess whether the full clinical-laboratory model added prognostic information beyond anatomical staging alone, we performed an additional TNM-only versus full-model comparison using the same imputation and cross-validation framework. In this internal analysis using GLM, the pooled cross-validated AUC increased from 0.548 (95% CI 0.45 to 0.65) for the TNM-only model to 0.668 (95% CI 0.55 to 0.77) for the full model. When AUCs were recomputed from the pooled cross-validated predictions, the AUC increased from 0.592 to 0.732, corresponding to an AUC difference of 0.140 (DeLong *p* = 0.0001). Reclassification metrics were also in favor of the full model (Integrated Discrimination Improvement IDI = 0.127; continuous Net Reclassification Index NRI = 0.801, with 0.535 for events and 0.266 for non-events). The full results are reported in the Supplementary Section “Additional full versus TNM-only predictive analysis”.

## 4. Discussion

The integration of machine learning (ML) approaches is increasingly used in oncology to improve risk stratification by modeling complex non-linear relationships that conventional regression models and TNM staging frequently overlook [4,19,20]. In this present study, six ML architectures were developed and internally validated to predict mortality risk in patients with bladder cancer (BC) using routinely collected clinicopathological and laboratory data.

In the present study, the pooled C-statistic was modest and broadly comparable across all six modeling approaches. The SVM achieved the highest point estimate (AUC 0.679, 95% CI 0.57 to 0.77), with only small differences compared with more conventional regression and tree-based methods. In clinical oncology, while a prognostic model is traditionally considered most robust when AUCs consistently exceed 0.75–0.80, contemporary validation efforts demonstrate that performance often varies significantly across independent patient populations [3,6]. For example, Dai et al. developed a machine-learning framework using the internal TCGA cohort, which yielded a high C-index ranging from 0.74 to 0.937. However, external validation revealed typical performance attenuation; when tested against independent datasets, the framework achieved a C-index range of 0.61 to 0.653 in the first validation cohort (GSE13507), followed by 0.53 to 0.598 in the second cohort (GSE31684), and 0.74 to 0.785 in the third cohort (GSE32894) [3]. Our observed value of 0.679 therefore represents modest but expected discrimination for a model based solely on readily accessible baseline clinical parameters that capture systemic host responses relevant to cancer outcomes. Furthermore, after determining the Youden-optimal threshold, sensitivity and specificity were relatively balanced across models, an operating profile also reported in several BC ML applications [7,9]. Nevertheless, choosing an operational threshold based on clinical practice remains preferred.

Beyond raw discrimination and classification, calibration analysis showed that our SVM model was relatively stable and well-calibrated, showing minimal divergence from the identity line and less variability across imputations than more flexible tree-based methods such as gradient boosting (GBM). In related BC ML work, calibration curves close to the diagonal and low Brier scores are used to formally demonstrate good calibration for boosting-based models [21,22]. Compared with the generalized additive model (GAM), SVM achieved slightly higher discrimination while preserving acceptable calibration and similar net benefit on decision curve analysis (DCA), suggesting that the kernel-based SVM may exploit complex, non-linear relationships in BC prognostic factors more efficiently than smoother-based approaches.

DCA further showed positive net benefit of the ML models compared to the reference strategies (classify all or none). Recent contemporary BC frameworks also utilize DCA alongside calibration curves and composite metrics to establish clinical utility beyond raw AUC and to guide optimal algorithm selection [21,22,23]. More broadly, DCA was specifically developed to evaluate whether prediction models provide net clinical benefit across threshold probabilities, complementing discrimination and calibration metrics [24].

To address missing laboratory data without losing power or introducing the biases inherent to complete-case analysis, we used Multiple Imputation by Chained Equations (MICE) to create 40 imputed datasets, allowing us to retain the full cohort (*N* = 337) and preserve the underlying multivariable structure of the data. MICE is widely recommended in medical research as an advanced MI technique that is superior to single imputation because it explicitly accounts for imputation uncertainty and operates by generating multiple completed datasets, analyzing each, and pooling results [25]. Empirical work on health indicators further shows that MICE maintains robust performance and preserves variance structure up to moderate levels of missingness, supporting its use for retaining sample size and multivariable relationships in longitudinal and clinical data [26]. In BC ML studies, multiple imputation has similarly been used to address missing clinicopathologic predictors before model fitting [21]. Although MPV missingness was found to be associated with the outcome in our dataset, the pooled variable importance (VIP) and SHAP values indicated limited impact on final predictions.

A notable finding from our modeling was that a systemic profile of blood and metabolic markers contributed more to predictions than traditional anatomical staging. This aligns with a growing body of evidence across various solid tumors, including colon, lung, and urologic malignancies, demonstrating that TNM staging alone frequently fails to capture prognostic heterogeneity, and that integrating additional clinical and biomarker features improves risk stratification [4,19,27]. In our cohort of 337 patients, this systemic profile, reflecting the host’s physiological response to the tumor, appeared to provide prognostic information not captured by anatomy alone, echoing observations that routine hematologic markers can refine risk groups beyond TNM in lung cancer [28].

Across our models, tumor grade was the strongest single predictor of outcome, consistent with its established importance in BC prognosis [6]. However, four of the five most important variables were routine blood tests: platelet count, bicarbonate (CO_2_), random glucose, and red blood cell count, all of which carried higher predictive weights than tumor stage in our machine learning models. Note that we distinguish between predictive relevance and causal inference when interpreting these algorithmic behaviors. Variable importance rankings and SHAP values quantify feature contributions to a model’s output on a given dataset and are now standard in BC ML, but are used purely as interpretability tools rather than causal estimators [21,22]. They therefore do not establish biological directionality, mechanistic causality, or intervention effects.

When interpreting these specific variables, they are best framed as surrogate measures of systemic inflammation, nutritional status, and metabolic stress rather than direct causal drivers. For example, the predictive effect of platelet count is congruent with recent evidence that machine learning-derived platelet-related signatures act as independent prognostic tools and reflect tumor biology and host immunity in BC and other solid tumors [29]; however, our findings do not imply that platelets directly cause tumor progression. Similarly, bicarbonate (CO_2_) and random glucose being among the top five features in terms of SHAP and VIP suggests that host–tumor metabolic disturbances may carry predictive weight. Hyperglycemia and metabolic syndrome components are associated with elevated BC risk [30]. Systemic acid–base disruptions and low bicarbonate levels have been prognostically linked to glycolysis-driven acidity and worse survival in non-small cell lung cancer [28]. In that context, our bicarbonate and glucose variables can be interpreted as statistical proxies of metabolic stress rather than direct evidence of a Warburg phenotype. Red blood cell-related measures also emerged as important predictors, consistent with meta-analytic data showing that indices such as RDW and anemia stratify cancer outcomes and reflect chronic inflammation or occult blood loss [31].

From a clinical perspective, the findings suggest that routinely measured laboratory markers contain prognostic information that is not fully captured by anatomical staging variables alone. Because our models utilize inexpensive, universally available, and routinely ordered blood parameters rather than high-cost multi-omics or advanced digital pathology pipelines, they represent a scalable framework for daily practice, similar in spirit to recent ML work integrating clinical and routine biomarker data to predict BC outcomes or metastasis [22]. Across the modeling approaches, tumor grade remained the strongest predictor, but several standard hematologic and metabolic markers, including platelet count, bicarbonate, random glucose, and red blood cell-related measures, were consistently prioritized. These variables are inexpensive, widely available, and already measured in routine care. Systematic and narrative reviews of AI deployment in BC emphasize that cost-effectiveness and immediate data availability are central to real-world clinical adoption, and that models leveraging routinely available clinical variables are more likely to be implementable than those requiring specialized assays [7,32]. However, this study may be interpreted as a proof-of-principle, and as a next step towards clinical deployment, external validation is required.

Despite the potential implications of these accessible parameters, several limitations must be explicitly acknowledged to place this study within the wider BC AI landscape. First, while our modeling utilized a rigorous stratified 10-fold cross-validation framework pooled across 40 multiple-imputed datasets to prevent information leakage, the study relies entirely on internal validation within a single-center cohort. Second, the retrospective design may introduce inherent selection biases and result in high baseline laboratory data missingness, which, although (partly) mitigated via MICE, introduces increased statistical uncertainty and potential bias. Third, the study omitted post-baseline treatment variables and event timings. Given that treatment selection and intensity are primary determinants of survival in BC, omitting these variables must be viewed as one of the main limitations. In addition, because follow-up was retrospective and not protocolized, the analysis used mortality occurrence during the study period rather than a formal time-to-event endpoint. These limitations should be addressed in future studies. Finally, our cohort features a heterogeneous mix of NMIBC and MIBC disease. The MIBC subgroup was small, with only 76 patients, and independent subgroup-specific model fitting would therefore have resulted in few events per predictor and unstable estimates. Consequently, the cross-validated AUC of 0.679 should be interpreted as an internally validated estimate for the overall cohort. As part of future work, performing separate performance analyses by disease subgroup would be beneficial.

## 5. Conclusions

In conclusion, our study demonstrates that integrating standard hematologic and metabolic blood markers into machine learning frameworks captures distinct systemic prognostic signals that incrementally enhance traditional anatomical staging frameworks in bladder cancer. While these routinely available clinical parameters offer a highly cost-effective substrate for risk stratification, the modest discriminative performance observed across all models underscores that they are not yet implementation-ready for standalone clinical practice. Because our findings are constrained by a retrospective, single-center design and a high proportion of baseline missing data, generalizability cannot be assumed. This work serves as an interpretable, data-driven proof of concept, rather than an operational tool. Prospective multicenter external validation, the integration of longitudinal post-baseline treatment variables, and fully powered sample sizes capable of robust subgroup analyses are mandatory next steps to establish true model transportability and define actionable clinical pathways.

## Figures and Tables

**Table 1:** Baseline characteristics of the study population overall and stratified by outcome. Continuous and categorical variables are summarized as median (IQR) and N (%). Group comparisons were performed using Wilcoxon rank-sum test and Fisher exact tests.

Covariate	Overall *N* = 337	Dead *N* = 228	Alive *N* = 109	*p*-Value
Gender				0.2
M	285 (85%)	197 (86%)	88 (81%)	
F	52 (15%)	31 (14%)	21 (19%)	
Diagnosis Age	64 (55, 72)	64 (55, 73)	63 (56, 72)	0.6
Age				>0.9
<65	181 (54%)	122 (54%)	59 (54%)	
>65	156 (46%)	106 (46%)	50 (46%)	
Overall Survival	21 (7, 51)	14 (3, 32)	43 (20, 81)	<0.001
Grade Of Tumor				<0.001
Low	116 (41%)	63 (34%)	53 (56%)	
High	166 (59%)	124 (66%)	42 (44%)	
Invasive	76 (23%)	56 (25%)	20 (18%)	0.2
Metastasis	47 (14%)	34 (15%)	13 (12%)	0.5
Papillary Tumor	195 (84%)	125 (82%)	70 (88%)	0.3
Tumor Stage				0.2
Early	61 (64%)	37 (60%)	24 (73%)	
Late	34 (36%)	25 (40%)	9 (27%)	
Cancer Recurrence	64 (39%)	40 (39%)	24 (38%)	>0.9
Lymph Node status	42 (35%)	29 (38%)	13 (29%)	0.3
Morphology				>0.9
TCC	267 (93%)	180 (93%)	87 (94%)	
SCC	12 (4.2%)	8 (4.1%)	4 (4.3%)	
ADC	7 (2.4%)	5 (2.6%)	2 (2.2%)	
Albumin	39.9 (37.0, 43.0)	40.0 (37.0, 42.0)	39.5 (37.5, 43.0)	0.7
MCH	28.1 (25.9, 30.0)	28.3 (25.6, 30.0)	27.9 (26.1, 29.7)	>0.9
CO_2_	22.0 (21.0, 24.0)	22.9 (21.0, 24.0)	22.0 (20.3, 24.0)	0.5
RBC	4.60 (4.00, 5.16)	4.60 (4.00, 5.20)	4.55 (4.10, 5.15)	>0.9
MCV	87 (81, 90)	87 (81, 91)	86 (81, 90)	0.6
RDW	13.80 (13.00, 15.45)	13.80 (13.00, 15.40)	13.93 (12.90, 15.80)	0.9
Glucose Random	6.9 (5.5, 10.1)	6.7 (5.4, 8.9)	7.3 (5.7, 10.9)	0.2
Globulin	30.0 (28.0, 35.0)	30.0 (29.0, 36.0)	29.5 (26.4, 33.5)	0.2
CRP	11 (4, 23)	13 (2, 26)	9 (5, 18)	>0.9
Ca	2.29 (2.22, 2.41)	2.35 (2.23, 2.42)	2.26 (2.21, 2.29)	0.2
Hemoglobin	13.10 (11.05, 14.60)	13.15 (10.98, 14.58)	13.10 (11.08, 15.00)	0.9
GGT	27 (18, 40)	32 (21, 51)	24 (18, 39)	0.2
PLT Count	268 (221, 320)	266 (223, 324)	274 (215, 303)	>0.9
MPV	7.95 (7.32, 8.85)	7.93 (6.88, 8.83)	7.95 (7.60, 8.90)	0.4
MCHC	32.50 (31.90, 33.70)	32.50 (31.70, 33.70)	32.80 (32.11, 33.83)	0.4
Total Protein	71 (67, 75)	72 (68, 75)	70 (67, 71)	0.5
Total Bilirubin	8 (6, 11)	8 (6, 11)	8 (7, 10)	0.8
Hematocrit	40 (34, 44)	40 (34, 44)	39 (34, 45)	0.7
PTT	30.0 (28.0, 32.0)	30.0 (26.5, 31.0)	30.5 (30.0, 34.0)	0.14
LDH	204 (186, 270)	210 (203, 277)	186 (182, 193)	0.2

**Table 2:** Pooled cross-validated model performance across six prediction approaches. Values are reported as point estimate (95% pooled confidence intervals) for AUC and threshold-based operating characteristics at a probability cutoff determined by the Youden index. The applied thresholds per model were 0.663, 0.655, 0.701, 0.687, 0.696, and 0.669.

METRIC (95% CI)	GLM	GLMNET	XGBOOST	GAM	GBM	SVM
POOLED AUC	0.662 (0.54–0.77)	0.671 (0.55–0.77)	0.663 (0.55–0.76)	0.653 (0.54–0.75)	0.639 (0.52–0.74)	0.679 (0.57–0.77)
SENSITIVITY	0.699 (0.36–0.91)	0.705 (0.43–0.88)	0.610 (0.35–0.82)	0.652 (0.39–0.84)	0.627 (0.36–0.83)	0.703 (0.48–0.86)
SPECIFICITY	0.603 (0.30–0.85)	0.603 (0.33–0.83)	0.680 (0.42–0.86)	0.631 (0.38–0.83)	0.637 (0.35–0.85)	0.601 (0.36–0.80)
POSITIVE PREDICTIVE VALUE	0.785 (0.68–0.86)	0.788 (0.69–0.86)	0.799 (0.69–0.88)	0.787 (0.68–0.86)	0.783 (0.68–0.86)	0.787 (0.69–0.86)
NEGATIVE PREDICTIVE VALUE	0.491 (0.33–0.65)	0.494 (0.35–0.64)	0.456 (0.33–0.59)	0.465 (0.33–0.61)	0.449 (0.33–0.57)	0.490 (0.36–0.62)
F1 SCORE	0.726 (0.53–0.86)	0.734 (0.59–0.84)	0.683 (0.50–0.82)	0.705 (0.55–0.82)	0.687 (0.51–0.82)	0.736 (0.62–0.83)

**Table 3:** Top five predictors by normalized variable importance (VIP) and normalized mean absolute SHAP values across six modeling approaches. The “Overall” column summarizes importance across methods. Variables marked with * are in both top 5.

Variable	GLM	GLMNET	XGB	GAM	GBM	SVM	Overall
VIP values							
Grade Of Tumor *	75.39	88.23	0.00	68.12	43.78	87.56	60.51
PLT Count *	73.42	0.30	67.78	0.20	81.08	74.12	49.48
CO_2_ *	57.93	6.78	54.79	4.08	51.38	64.84	39.97
Glucose Random	41.02	3.31	51.51	2.11	55.31	36.40	31.61
RBC *	44.73	29.89	32.77	14.28	32.41	17.17	28.54
SHAP values							
Grade Of Tumor *	0.0927	0.1102	0.2032	0.0974	0.1063	0.1320	0.1236
PLT Count *	0.0895	0.1040	0.1525	0.1048	0.1388	0.1252	0.1191
RBC *	0.0854	0.0857	0.0708	0.0756	0.0663	0.0824	0.0777
CO_2_ *	0.0470	0.0532	0.1492	0.0533	0.0613	0.0651	0.0715
MCV	0.0904	0.0714	0.0386	0.0841	0.0404	0.0476	0.0621

## Data Availability

The datasets used during the current study are in the article. Further inquiries can be directed to the corresponding author. R 4.5.3 and Python 3.12 software to run the complete imputation and ML pipeline is publicly available and can be found under https://github.com/selbouhaddani/ML4BladderCancerEHR_Diagnostics_MDPI (accessed on 25 June 2026).

## Supplementary Materials

The following supporting information can be downloaded at: https://www.mdpi.com/article/10.3390/diagnostics16213486/s1, Supplementary tables and figures supporting the analyses in the main manuscript: (1) full SHAP and VIP analyses, (2) Missingness analyses and demographic patterns, (3) Hyperparameter search strategy, (4) Additional full versus TNM-only predictive analysis.

## Abbreviations

The following abbreviations are used in this manuscript:

ML	Machine Learning
BC	Bladder Cancer
AUC	Area Under the Receiver
DCA	Decision Curve Analysis
GLM	Logistic Regression
GLMNET	Elastic Net Logistic Regression
GAM	Generalized Additive Models
XGBoost	Extreme Gradient Boosting
GBM	Gradient Boosting Machines
SVM	Support Vector Machines

## References

[1] López-Beltran A., Cookson M., Guercio B., Cheng L. (2024). Advances in diagnosis and treatment of bladder cancer. BMJ.

[2] Kiruba B., Narayan P., Raj B., Raj S., Mathew S.G., Lulu S.S., Sundararajan V. (2025). Intervention of machine learning in bladder cancer research using multi-omics datasets: Systematic review on biomarker identification. Discov. Oncol..

[3] Dai H., Yu Z., Zhao Y., Jiang K., Hang Z., Huang X., Ma H., Wang L., Li Z., Wu M. (2025). Integrating machine learning models with multi-omics analysis to decipher the prognostic significance of mitotic catastrophe heterogeneity in bladder cancer. Biol. Direct.

[4] Brieu N., Gavriel C., Nearchou I., Harrison D., Schmidt G., Caie P. (2019). Automated tumour budding quantification by machine learning augments TNM staging in muscle-invasive bladder cancer prognosis. Sci. Rep..

[5] Darrell C., Montironi R., Paner G. (2020). Potential biomarkers and risk assessment models to enhance the tumor-node-metastasis (TNM) staging classification of urologic cancers. Expert Rev. Mol. Diagn..

[6] Chen S., Jiang L., Zheng X., Shao J., Wang T., Zhang E., Gao F., Wang X., Zheng J. (2021). Clinical use of machine learning-based pathomics signature for diagnosis and survival prediction of bladder cancer. Cancer Sci..

[7] Abbas S., Shafik R., Soomro N., Heer R., Adhikari K. (2025). AI predicting recurrence in non-muscle-invasive bladder cancer: Systematic review with study strengths and weaknesses. Front. Oncol..

[8] Kwong J., Wu J., Malik S., Khondker A., Gupta N., Bodnariuc N., Narayana K., Malik M., Van Der Kwast T., Johnson A. (2024). Predicting non-muscle invasive bladder cancer outcomes using artificial intelligence: A systematic review using APPRAISE-AI. npj Digit. Med..

[9] Gavriel C., Dimitriou N., Brieu N., Nearchou I., Arandjelovíc O., Schmidt G., Harrison D., Caie P. (2021). Assessment of Immunological Features in Muscle-Invasive Bladder Cancer Prognosis Using Ensemble Learning. Cancers.

[10] Khoraminia F., Fuster S., Kanwal N., Olislagers M., Engan K., Van Leenders G., Stubbs A., Akram F., Zuiverloon T. (2023). Artificial Intelligence in Digital Pathology for Bladder Cancer: Hype or Hope? A Systematic Review. Cancers.

[11] Bashkami A., Nasayreh A., Makhadmeh S., Gharaibeh H., Alzahrani A., Alwadain A., Jia H., Absalom E., Abualigah L. (2024). A review of Artificial Intelligence methods in bladder cancer: Segmentation, classification, and detection. Artif. Intell. Rev..

[12] Ramberger E., Standvoss K., Ghosh S., Haegele M., Eich M.-L., Anders P., Tharun L., Moellers A., Dernbach G., Ribbat-Idel J. (2025). Enhancing prognostic precision in bladder cancer: AI-driven tumor microenvironment analysis from H&E images. J. Clin. Oncol..

[13] Wang H.-X., Zhang M., Miao J., Hou F., Chen Y., Huang Y., Yang L., Yang S., Huang C., Song Y. (2023). Deep learning signature based on multiphase enhanced CT for bladder cancer recurrence prediction: A multi-center study. eClinicalMedicine.

[14] Yan H., Ji X., Li B. (2025). Advancing personalized, predictive, and preventive medicine in bladder cancer: A multi-omics and machine learning approach for novel prognostic modeling, immune profiling, and therapeutic target discovery. Front. Immunol..

[15] Smani S., DuBois J., Zhao K., Sutherland R., Rahman S.N., Humphrey P., Hesse D., Tan W.S., Martin D., Lokeshwar S.D. (2025). Advancements in the Diagnosis, Treatment, and Risk Stratification of Non-Muscle Invasive Bladder Cancer. Curr. Oncol. Rep..

[16] Teng F., Zhang R., Wang Y., Li Q., Wang B., Chen H., Liu T., Liu Z., Meng J., Dong S. (2025). Machine Learning and Mendelian Randomization Reveal a Tumor Immune Cell Profile for Predicting Bladder Cancer Risk and Immunotherapy Outcomes. Am. J. Pathol..

[17] Chen W., Li Z., Liu L., Heidari A., Cai Z., Chen H., Li J. (2025). An intelligent system for the diagnosis of bladder cancer using enhanced hunger games search and support vector machine. Biomed. Signal Process. Control..

[18] Shen H., Zheng F., Tang X., Ma Z., Tao X., Deng Z., Yao Z. (2025). Constructing a bladder cancer prognostic model related to exosome using machine learning and identifying THBS1 as a potential target. Cancer Cell Int..

[19] Hueman M., Wang H., Liu Z., Henson D., Nguyen C., Park D., Sheng L., Chen D. (2021). Expanding TNM for lung cancer through machine learning. Thorac. Cancer.

[20] Gupta P., Chiang S.-F., Sahoo P., Mohapatra S., You J.-F., Onthoni D.D., Hung H.-Y., Chiang J., Huang Y., Tsai W. (2019). Prediction of Colon Cancer Stages and Survival Period with Machine Learning Approach. Cancers.

[21] Dai L., Ye K., Yao G., Lin J., Tan Z., Wei J.-Q., Hu Y., Luo J., Fang Y., Chen W. (2025). Using machine learning for predicting cancer-specific mortality in bladder cancer patients undergoing radical cystectomy: A SEER-based study. BMC Cancer.

[22] Deng Q., Li S., Zhang Y., Jia Y., Yang Y. (2025). Development and validation of interpretable machine learning models to predict distant metastasis and prognosis of muscle-invasive bladder cancer patients. Sci. Rep..

[23] Wang B., Gong Z., Su P., Zhen G., Zeng T., Ye Y. (2025). Multi-machine learning model based on radiomics features to predict prognosis of muscle-invasive bladder cancer. BMC Cancer.

[24] Vickers A.J., Holland F. (2021). Decision curve analysis to evaluate the clinical benefit of prediction models. Spine J..

[25] Bao W. (2016). Multiple imputation with multivariate imputation by chained equation (MICE) package. Ann. Transl. Med..

[26] Junaid K., Kiran T., Gupta M., Kishore K., Siwatch S. (2025). How much missing data is too much to impute for longitudinal health indicators? A preliminary guideline for the choice of the extent of missing proportion to impute with multiple imputation by chained equations. Popul. Health Metr..

[27] Cotler J., Janczewski L., Weigel R., Zhu X., Chan K., Palis B., Edge S., Nelson H., Boughey J. (2025). Beyond TNM staging: Machine learning of individualized colon cancer outcomes. Surgery.

[28] Ion-Andrei D.-G., Gache A.-C., Mocanu E., Postu A.-C., Lupșă S.-A., Mocanu L., Muntean C., Danteș E. (2025). Prognostic Value of Routine Hematological Markers and ECOG Performance in Predicting Overall Survival in Lung Cancer—A Retrospective Cohort Study and Literature Review. J. Clin. Med..

[29] Chen C., Zhang J., Liu X., Zhuang Q., Lu H., Hou J. (2024). A platelet-related signature for predicting the prognosis and immunotherapy benefit in bladder cancer based on machine learning combinations. Transl. Androl. Urol..

[30] Ahmadinezhad M., Arshadi M., Hesari E., Sharafoddin M., Azizi H., Khodamoradi F. (2022). The relationship between metabolic syndrome and its components with bladder cancer: A systematic review and meta-analysis of cohort studies. Epidemiol. Health.

[31] Ai L., Mu S., Hu Y. (2018). Prognostic role of RDW in hematological malignancies: A systematic review and meta-analysis. Cancer Cell Int..

[32] Borhani S., Borhani R., Kajdacsy-Balla A. (2022). Artificial intelligence: A promising frontier in bladder cancer diagnosis and outcome prediction. Crit. Rev. Oncol. Hematol..

