# Supplementary material for: From Stage to Systems: Integrating Standard Laboratory Markers into Bladder Cancer Prognostic Models
Source: Diagnostics (Basel). 2026 Oct 24;16(21):3486. doi: 10.3390/diagnostics16213486 (PMC13762294; doi:10.3390/diagnostics16213486)
Supplement: Supplementary file 1 [file diagnostics-16-03486-s001.zip › diagnostics-4345501-supplementary.pdf]

Supplement to “*From Stage to Systems: Integrating Standard Laboratory Markers into Bladder Cancer Prognostic Models*”

Table of Contents

Full SHAP and Variable Importance analyses 2

SHAP beeswarm plots 2

Aggregated VIP and SHAP values per predictor and method 4

Missingness analyses and demographic patterns 5

Missingness patterns by outcome 5

Missingness patterns by Gender 7

Missingness patterns by diagnosis age (median split) 9

Hyperparameter search strategy 11

Additional full versus TNM-only predictive analysis 12

Main AUC metric: pooled AUCs 12

Comparison of AUCs of pooled predictions 12

Full SHAP and Variable Importance analyses

SHAP beeswarm plots

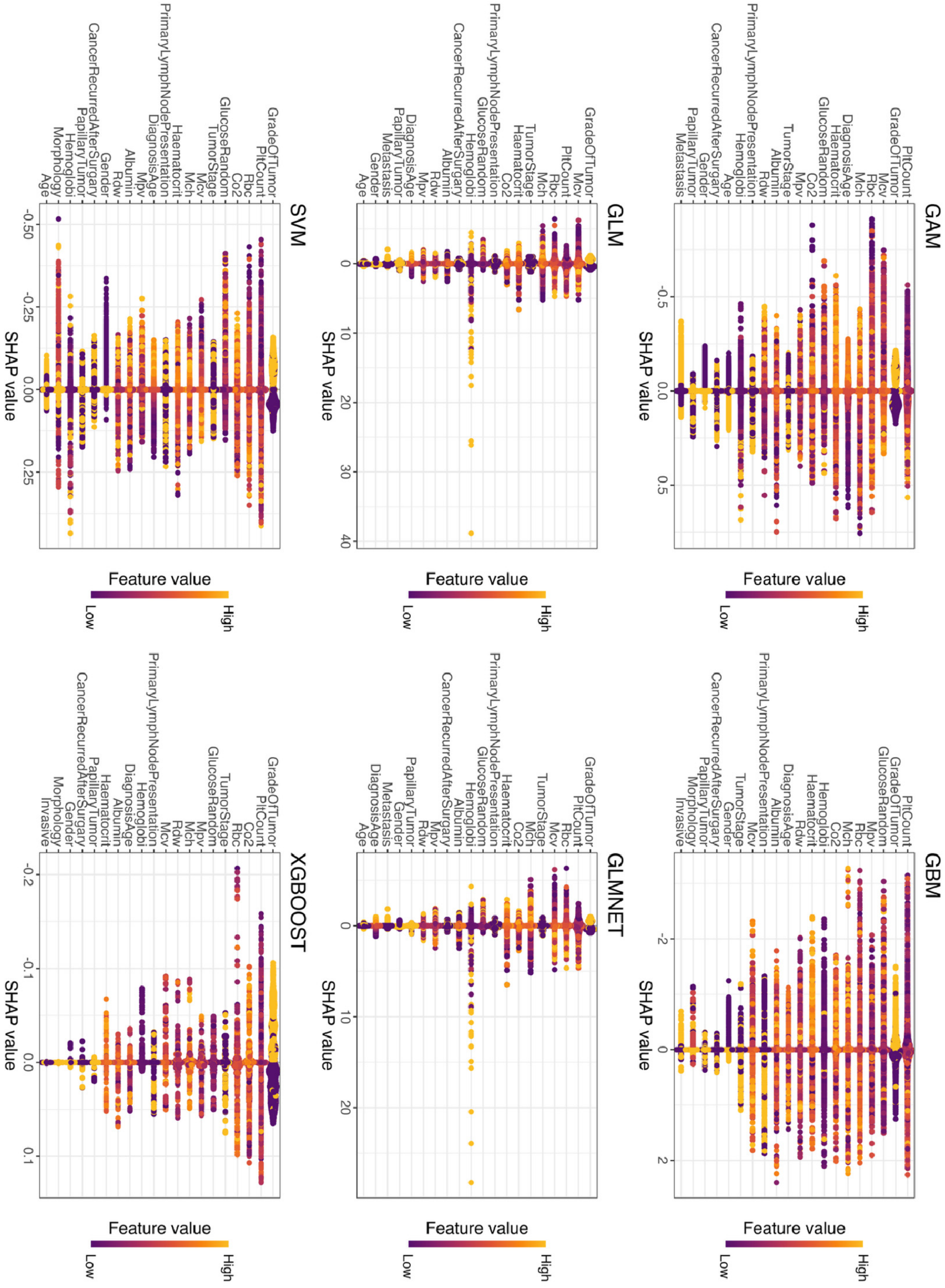

Figure S1. Individual SHAP values per predictor and modelling approach.

Aggregated VIP and SHAP values per predictor and method

Table S1. Normalized variable importance (VIP) and normalized mean absolute SHAP values across six modelling approaches for all predictors. The "Overall" column summarizes importance across methods.

Variable	glm	glmnet	xgb	gam	gbm	svm	Overall
**VIP values**							
**GradeOfTumor***	75.39	88.23	0.00	68.12	43.78	87.56	60.51
**PltCount***	73.42	0.30	67.78	0.20	81.08	74.12	49.48
**Co2**	57.93	6.78	54.79	4.08	51.38	64.84	39.97
**GlucoseRandom**	41.02	3.31	51.51	2.11	55.31	36.40	31.61
**Rbc***	44.73	29.89	32.77	14.28	32.41	17.17	28.54
**TumorStage**	51.79924	38.684908	0	25.297091	11.8452118	37.924215	27.591779
**Mpv**	28.49875	5.2329389	34.68186	3.4545927	38.4741542	28.827504	23.194967
**Diagnosis Age**	11.56626	0.158348	28.71996	57.2232291	30.4113269	5.69362	22.295457
**Hemoglobin**	30.39886	2.6136592	30.5019	1.4576124	37.957366	20.053636	20.497172
**Rdw**	20.10042	2.162725	33.62886	1.6697552	30.854860	22.994943	18.568595
**PrimaryLymphNodePresentation**	34.47065	24.130656	0	12.12563	4.737738	32.980862	18.074256
**Haematocrit**	32.58222	1.280831	22.94843	0.7431375	34.545640	15.976271	18.012755
**Mch**	24.50193	4.2458026	28.50756	2.0824066	27.696206	15.794294	17.138033
**Morphology**	29.68704	40.387409	0	22.443462	0.2263742	7.649743	16.732338
**PapillaryTumor**	31.8594	24.195392	0	11.577547	3.4457781	28.697444	16.62926
**Albumin**	26.78789	1.2951172	14.78262	0.5638442	26.1631097	29.927913	16.586749
**Mcv**	29.11346	1.6711597	15.32441	0.7397006	23.4625859	14.265874	14.096198
**Invasive**	22.80428	14.231689	0	7.6733777	1.4025108	24.245587	11.72624
**Gender**	20.52042	14.100474	0	7.5308523	4.351281	22.011324	11.419059
**Metastasis**	23.39989	16.081953	0	9.2840849	0.4764785	10.972407	10.035803
**CancerRecurredAfterSurgary**	20.28679	11.342219	0	6.1102005	2.2062372	15.089227	9.172446
**Age**	16.064	8.1078432	0	6.1619456	0.3476391	1.240948	5.320395
**SHAP values**							
**GradeOfTumor**	0.0927	0.1102	0.0974	0.1063	0.132	0.2032	0.1236

Variable	glm	glmnet	xgb	gam	gbm	svm	Overall
**PltCount**	0.0895	0.104	0.1048	0.1388	0.1252	0.1525	0.1191
**Rbc**	0.0854	0.0857	0.0756	0.0663	0.0824	0.0708	0.0777
**Co2**	0.047	0.0532	0.0533	0.0613	0.0651	0.1492	0.0715
**Mcv**	0.0904	0.0714	0.0841	0.0404	0.0476	0.0386	0.0621
**Mch**	0.0787	0.0646	0.0749	0.0649	0.0469	0.0397	0.0616
**GlucoseRandom**	0.0365	0.042	0.0623	0.0857	0.0611	0.0484	0.056
**TumorStage**	0.0656	0.0655	0.048	0.0275	0.0574	0.0692	0.0556
**Haematocrit**	0.0529	0.0482	0.0631	0.0538	0.0463	0.0193	0.0473
**Mpv**	0.0302	0.0317	0.0483	0.0677	0.034	0.0419	0.0423
**PrimaryLymphNodePresentation**	0.0459	0.0449	0.0291	0.0408	0.0413	0.036	0.0397
**Diagnosis Age**	0.0253	0.0228	0.0711	0.0459	0.038	0.0257	0.0381
**Rdw**	0.0303	0.0291	0.0325	0.0496	0.028	0.0396	0.0348
**Hemoglobin**	0.0353	0.035	0.0268	0.0596	0.0202	0.0258	0.0338
**Albumin**	0.0312	0.0325	0.033	0.042	0.0309	0.023	0.0321
**CancerRecurredAfterSurgary**	0.0334	0.0323	0.0166	0.0088	0.0275	0.0051	0.0206
**Gender**	0.0228	0.0252	0.016	0.0166	0.0278	0.0035	0.0186
**PapillaryTumor**	0.0247	0.0258	0.012	0.0082	0.0241	0.0054	0.0167
**Age**	0.0225	0.0185	0.0242	0.0012	0.0175	0.0007	0.0141
**Metastasis**	0.0236	0.0235	0.0112	0.0045	0.0156	0.0007	0.0132
**Morphology**	0.0175	0.0175	0.009	0.0056	0.0183	0.0008	0.0115
**Invasive**	0.0187	0.0166	0.0067	0.0046	0.0129	0.0008	0.01

Missingness analyses and demographic patterns

Missingness patterns by outcome

Table S2. Missingness patterns by outcome status.

**Missingness indicator**	**Overall** (N = 337)	**Dead** (N = 228)	**Alive** (N = 109)	**p-value**	**q-value**
DiagnosisAge				0.10	0.8
Observed	330 (98%)	221 (97%)	109 (100%)		
Missing	7 (2.1%)	7 (3.1%)	0 (0%)		

**Missingness indicator**	**Overall** (N = 337)	**Dead** (N = 228)	**Alive** (N = 109)	**p-value**	**q-value**
OverallSurvival				>0.9	>0.9
Observed	336 (100%)	227 (100%)	109 (100%)		
Missing	1 (0.3%)	1 (0.4%)	0 (0%)		
GradeOfTumor				0.3	>0.9
Observed	282 (84%)	187 (82%)	95 (87%)		
Missing	55 (16%)	41 (18%)	14 (13%)		
PapillaryTumor				0.3	>0.9
Observed	233 (69%)	153 (67%)	80 (73%)		
Missing	104 (31%)	75 (33%)	29 (27%)		
TumorStage				0.6	>0.9
Observed	95 (28%)	62 (27%)	33 (30%)		
Missing	242 (72%)	166 (73%)	76 (70%)		
CancerRecurredAfterSurgary				0.036	0.5
Observed	166 (49%)	103 (45%)	63 (58%)		
Missing	171 (51%)	125 (55%)	46 (42%)		
PrimaryLymphNodePresentation				0.2	>0.9
Observed	121 (36%)	76 (33%)	45 (41%)		
Missing	216 (64%)	152 (67%)	64 (59%)		
Morphology				>0.9	>0.9
Observed	286 (85%)	193 (85%)	93 (85%)		
Missing	51 (15%)	35 (15%)	16 (15%)		
Albumin				0.022	0.4
Observed	122 (36%)	73 (32%)	49 (45%)		
Missing	215 (64%)	155 (68%)	60 (55%)		
Mch				0.027	0.4
Observed	171 (51%)	106 (46%)	65 (60%)		
Missing	166 (49%)	122 (54%)	44 (40%)		
Co2				0.073	0.7
Observed	78 (23%)	46 (20%)	32 (29%)		
Missing	259 (77%)	182 (80%)	77 (71%)		
Rbc				0.047	0.6

Missingness indicator	**Overall** (N = 337)	**Dead** (N = 228)	**Alive** (N = 109)	**p-value**	**q-value**
Observed	164 (49%)	102 (45%)	62 (57%)		
Missing	173 (51%)	126 (55%)	47 (43%)		
Mcv				0.048	0.6
Observed	171 (51%)	107 (47%)	64 (59%)		
Missing	166 (49%)	121 (53%)	45 (41%)		
Rdw				0.3	>0.9
Observed	153 (45%)	99 (43%)	54 (50%)		
Missing	184 (55%)	129 (57%)	55 (50%)		
GlucoseRandom				0.010	0.2
Observed	145 (43%)	87 (38%)	58 (53%)		
Missing	192 (57%)	141 (62%)	51 (47%)		
Hemoglobin				0.062	0.6
Observed	166 (49%)	104 (46%)	62 (57%)		
Missing	171 (51%)	124 (54%)	47 (43%)		
PltCount				0.032	0.4
Observed	71 (21%)	40 (18%)	31 (28%)		
Missing	266 (79%)	188 (82%)	78 (72%)		
Mpv				<0.001	0.015
Observed	109 (32%)	60 (26%)	49 (45%)		
Missing	228 (68%)	168 (74%)	60 (55%)		
Haematocrit				0.015	0.2
Observed	171 (51%)	105 (46%)	66 (61%)		
Missing	166 (49%)	123 (54%)	43 (39%)		

Missingness patterns by Gender

Table S3. Missingness patterns by gender.

**Missingness indicator**	**Overall** (N = 337)	**M** (N = 285)	**F** (N = 52)	**p-value**	**q-value**
DiagnosisAge				>0.9	>0.9
Observed	330 (98%)	279 (98%)	51 (98%)		
Missing	7 (2.1%)	6 (2.1%)	1 (1.9%)		
OverallSurvival				0.2	>0.9
Observed	336 (100%)	285 (100%)	51 (98%)		
Missing	1 (0.3%)	0 (0%)	1 (1.9%)		
GradeOfTumor				0.8	>0.9
Observed	282 (84%)	239 (84%)	43 (83%)		
Missing	55 (16%)	46 (16%)	9 (17%)		
PapillaryTumor				0.071	>0.9
Observed	233 (69%)	203 (71%)	30 (58%)		
Missing	104 (31%)	82 (29%)	22 (42%)		
TumorStage				0.6	>0.9
Observed	95 (28%)	82 (29%)	13 (25%)		
Missing	242 (72%)	203 (71%)	39 (75%)		
CancerRecurredAfterSurgary				0.10	>0.9
Observed	166 (49%)	146 (51%)	20 (38%)		
Missing	171 (51%)	139 (49%)	32 (62%)		
PrimaryLymphNodePresentation				0.041	0.7
Observed	121 (36%)	109 (38%)	12 (23%)		
Missing	216 (64%)	176 (62%)	40 (77%)		
Morphology				0.037	0.7
Observed	286 (85%)	247 (87%)	39 (75%)		
Missing	51 (15%)	38 (13%)	13 (25%)		
Albumin				0.2	>0.9
Observed	122 (36%)	99 (35%)	23 (44%)		
Missing	215 (64%)	186 (65%)	29 (56%)		
Mch				0.051	0.9
Observed	171 (51%)	138 (48%)	33 (63%)		
Missing	166 (49%)	147 (52%)	19 (37%)		
Co2				0.076	>0.9
Observed	78 (23%)	71 (25%)	7 (13%)		
Missing	259 (77%)	214 (75%)	45 (87%)		
Rbc				0.2	>0.9
Observed	164 (49%)	134 (47%)	30 (58%)		
Missing	173 (51%)	151 (53%)	22 (42%)		
Mcv				0.2	>0.9
Observed	171 (51%)	140 (49%)	31 (60%)		
Missing	166 (49%)	145 (51%)	21 (40%)		
Rdw				0.5	>0.9
Observed	153 (45%)	127 (45%)	26 (50%)		
Missing	184 (55%)	158 (55%)	26 (50%)		
GlucoseRandom				0.2	>0.9
Observed	145 (43%)	118 (41%)	27 (52%)		
Missing	192 (57%)	167 (59%)	25 (48%)		

**Missingness indicator**	**Overall** (N = 337)	**M** (N = 285)	**F** (N = 52)	**p-value**	**q-value**
Hemoglobin				0.070	>0.9
Observed	166 (49%)	134 (47%)	32 (62%)		
Missing	171 (51%)	151 (53%)	20 (38%)		
PltCount				0.2	>0.9
Observed	71 (21%)	64 (22%)	7 (13%)		
Missing	266 (79%)	221 (78%)	45 (87%)		
Mpv				0.15	>0.9
Observed	109 (32%)	97 (34%)	12 (23%)		
Missing	228 (68%)	188 (66%)	40 (77%)		
Haematocrit				0.10	>0.9
Observed	171 (51%)	139 (49%)	32 (62%)		
Missing	166 (49%)	146 (51%)	20 (38%)		

Missingness patterns by diagnosis age (median split)

Table S4. Missingness patterns by diagnosis age, split at median (64 years). Note that the prediction models use the full continuous diagnosis age.

**Missingness indicator**	**Overall** (N = 330)	**<=64** (N = 174)	**>64** (N = 156)	**p-value**	**q-value**
DiagnosisAge				>0.9	>0.9
Observed	330 (100%)	174 (100%)	156 (100%)		
Missing	0 (0%)	0 (0%)	0 (0%)		
OverallSurvival				0.5	>0.9
Observed	329 (100%)	174 (100%)	155 (99%)		
Missing	1 (0.3%)	0 (0%)	1 (0.6%)		
GradeOfTumor				0.8	>0.9
Observed	280 (85%)	149 (86%)	131 (84%)		
Missing	50 (15%)	25 (14%)	25 (16%)		
PapillaryTumor				0.7	>0.9
Observed	230 (70%)	123 (71%)	107 (69%)		
Missing	100 (30%)	51 (29%)	49 (31%)		
TumorStage				0.3	>0.9
Observed	95 (29%)	55 (32%)	40 (26%)		
Missing	235 (71%)	119 (68%)	116 (74%)		
CancerRecurredAfterSurgary				0.2	>0.9
Observed	162 (49%)	79 (45%)	83 (53%)		
Missing	168 (51%)	95 (55%)	73 (47%)		

**Missingness indicator**	**Overall** (N = 330)	**<=64** (N = 174)	**>64** (N = 156)	**p-value**	**q-value**
PrimaryLymphNodePresentation				0.6	>0.9
Observed	119 (36%)	65 (37%)	54 (35%)		
Missing	211 (64%)	109 (63%)	102 (65%)		
Morphology				0.6	>0.9
Observed	283 (86%)	151 (87%)	132 (85%)		
Missing	47 (14%)	23 (13%)	24 (15%)		
Albumin				0.4	>0.9
Observed	121 (37%)	60 (34%)	61 (39%)		
Missing	209 (63%)	114 (66%)	95 (61%)		
Mch				0.8	>0.9
Observed	171 (52%)	89 (51%)	82 (53%)		
Missing	159 (48%)	85 (49%)	74 (47%)		
Co2				0.2	>0.9
Observed	78 (24%)	46 (26%)	32 (21%)		
Missing	252 (76%)	128 (74%)	124 (79%)		
Rbc				>0.9	>0.9
Observed	163 (49%)	86 (49%)	77 (49%)		
Missing	167 (51%)	88 (51%)	79 (51%)		
Mcv				>0.9	>0.9
Observed	170 (52%)	89 (51%)	81 (52%)		
Missing	160 (48%)	85 (49%)	75 (48%)		
Rdw				>0.9	>0.9
Observed	152 (46%)	80 (46%)	72 (46%)		
Missing	178 (54%)	94 (54%)	84 (54%)		
GlucoseRandom				0.6	>0.9
Observed	144 (44%)	73 (42%)	71 (46%)		
Missing	186 (56%)	101 (58%)	85 (54%)		
Hemoglobin				0.7	>0.9
Observed	165 (50%)	85 (49%)	80 (51%)		
Missing	165 (50%)	89 (51%)	76 (49%)		
PltCount				0.083	>0.9

**Missingness indicator**	**Overall** (N = 330)	**<=64** (N = 174)	**>64** (N = 156)	**p-value**	**q-value**
Observed	71 (22%)	44 (25%)	27 (17%)		
Missing	259 (78%)	130 (75%)	129 (83%)		
Mpv				0.3	>0.9
Observed	108 (33%)	61 (35%)	47 (30%)		
Missing	222 (67%)	113 (65%)	109 (70%)		
Haematocrit				0.7	>0.9
Observed	170 (52%)	88 (51%)	82 (53%)		
Missing	160 (48%)	86 (49%)	74 (47%)		

Hyperparameter search strategy

Table S5. Final hyperparameter grid for all prediction models

Method	Hyperparameter	Range
Elastic net (GLMNET)	l1_ratio (alpha)	0 to 1 in steps of 0.1
Elastic net (GLMNET)	C (inverse penalty)	logspace 10^-0.3 to 10^4 (25 values)
XGBoost	max_depth	{2, 3, 4, 5}
XGBoost	eta (learning rate)	0.10 to 0.20 in steps of 0.02
XGBoost	gamma	{0, 0.001, 0.01, 0.1}
XGBoost	num_rounds (early stop)	up to 300, early stopping 50
GBM	n_estimators	100 to 1700 (10 values)
GBM	learning_rate	0.0005 plus 0.003 to 0.05 (10 values)
GBM	max_depth	2 (fixed), min_samples_leaf = 10
SVM (RBF)	C	2^-1 to 2^7
SVM (RBF)	gamma	2^-10 to 2^-3 (step 2)
GAM	spline basis + elastic net	cubic B-spline, 5 knots, on continuous predictors; enet grid as GLMNET

Additional full versus TNM-only predictive analysis

Main AUC metric: pooled AUCs

Table S6. Pooled cross-validated AUCs comparing full and TNM only models

model	95% Low	C-statistic	95% Up
Full (GLM)	0.5524	0.6680	0.7664
TNM-only (GLM)	0.4475	0.5484	0.6454

Comparison of AUCs of pooled predictions

Table S7. AUCs based on pooled cross-validated predictions comparing full and TNM only models.

Quantity	Value
AUC full (pooled pred.)	0.732
AUC TNM-only (pooled pred.)	0.592
AUC difference	0.140
DeLong p-value	0.0001
IDI	0.1267
Continuous NRI	0.8011
NRI events	0.5351
NRI non-events	0.2661

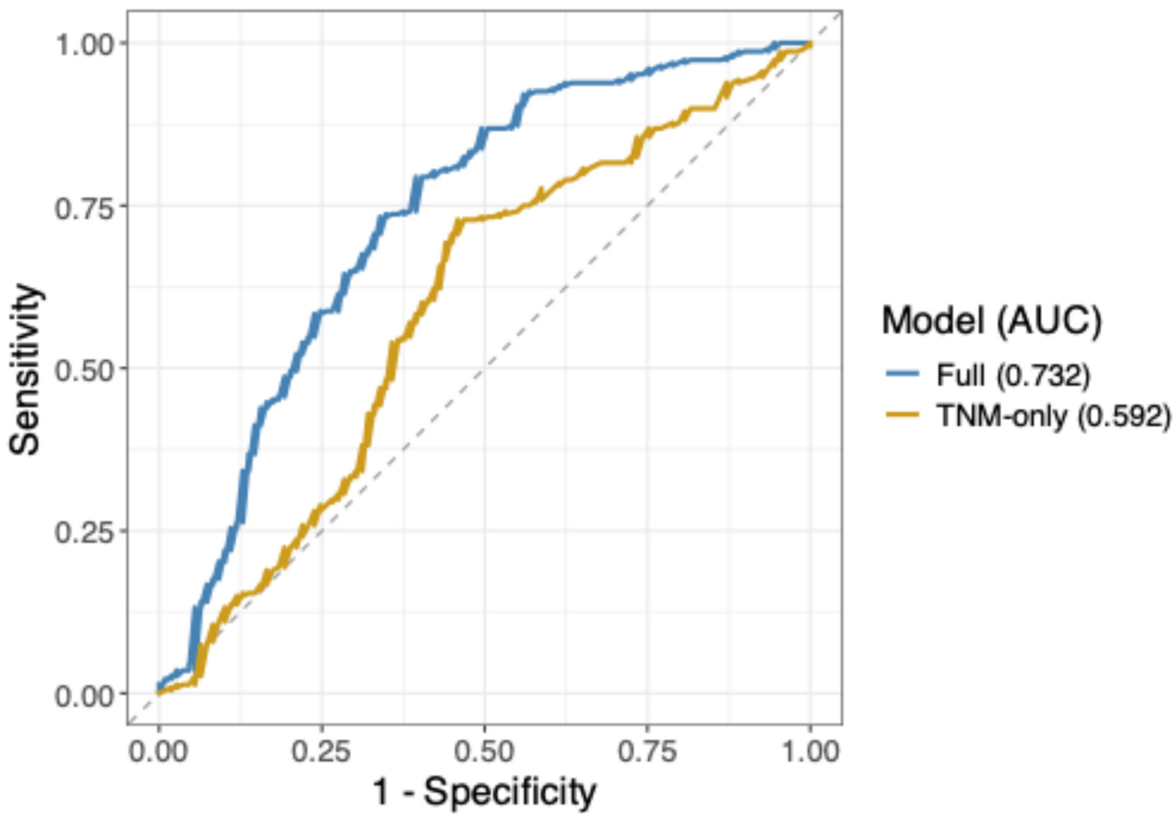

Figure S2. ROC based on pooled cross-validated predictions comparing full and TNM only models.
